# Uterus Didelphys Bicollis Diagnosed Intraoperatively in a Primigravida After Long-Standing Primary Infertility: A Case Report

**DOI:** 10.7759/cureus.116537

**Published:** 2026-09-19

**Authors:** Gareema Naik, Dhvani Rolekar, Miteshkumar H Kaklotar, Yuktaa Naik

**Affiliations:** 1 Obstetrics and Gynaecology, G.M.E.R.S. Medical College and Hospital, Valsad, Valsad, IND; 2 Obstetrics and Gynaecology, Krishna Institute of Medical Sciences Deemed University, Karad, IND

**Keywords:** intraoperative diagnosis, müllerian anomaly, pelvic examination, primary infertility, uterus didelphys

## Abstract

Uterus didelphys is a rare Müllerian duct anomaly, characterized by complete duplication of the uterine cavity and cervix. It is often associated with a longitudinal vaginal septum. Patients may present with dysmenorrhea, dyspareunia, and abnormal uterine bleeding, although it may remain asymptomatic until pregnancy. We report a case of a 41-year-old primigravida with 22 years of primary infertility, diagnosed with uterus didelphys intraoperatively. She presented with intermittent per-vaginal spotting and lower abdominal enlargement. Transabdominal ultrasonography (USG) revealed an unexpected 20-week pregnancy. Despite multiple subsequent antenatal ultrasonographic examinations, the uterine anomaly was not identified. At 32 weeks and 6 days, she presented with lower abdominal pain, and USG revealed severe oligohydramnios, breech presentation, and asymmetric intrauterine growth restriction. Per-vaginal examination revealed a longitudinal vaginal septum with two vaginal canals, each leading into a separate cervix. An emergency lower-segment caesarean section (LSCS) was performed. Intraoperatively, the uterus was found to consist of two separate uterine cavities that were not communicating, each leading into a distinct cervix, consistent with uterus didelphys bicollis​​​​​​. Uterus didelphys bicollis was confirmed. A live male infant weighing 1.810 kg was delivered. Both the mother and neonate recovered uneventfully. This case highlights that uterus didelphys may remain undiagnosed despite prolonged infertility and multiple antenatal USG. A thorough pelvic examination may provide an important clinical clue and prompt targeted imaging. Early diagnosis of Müllerian anomalies may support individualized counselling, appropriate imaging, and risk-based obstetric surveillance.

## Introduction

The female reproductive tract, including the uterus, fallopian tubes, and cervix, develops from the Müllerian (paramesonephric) ducts, which also contribute to the upper vagina. During normal embryogenesis, the paired Müllerian ducts extend caudally, with their cranial portions forming the fallopian tubes, whereas the caudal portions fuse, with resorption of the intervening septum, leading to formation of a single uterine cavity and cervix. Failure of this fusion or septal resorption results in an abnormal reproductive tract anatomy, collectively known as congenital Müllerian Duct Anomalies (MDAs) [1,2]. These uterine malformations are associated with increased risk of subfertility or infertility, menstrual abnormalities, recurrent pregnancy loss, and obstetric complications such as prematurity, malpresentation, caesarean section, placental abruption, and fetal growth restriction (FGR) [2-6]. However, outcomes are highly variable depending on the type of anomaly [1].

Congenital uterine anomalies affect up to 5.5% to 6.7% of the general female population [4,5]. Uterus didelphys represents roughly 8% of all the congenital uterine anomalies, affecting almost 0.3% of women in the general population [1]. It is characterized by two distinct uterine cavities, each with its own cervix, and often a longitudinal vaginal septum may coexist. In the historical American Fertility Society (AFS) classification, uterus didelphys was a Class III uterine anomaly; however, the current American Society for Reproductive Medicine Müllerian Anomalies Classification 2021 (ASRM MAC2021) uses descriptive terminology rather than numbered classes and recognizes uterus didelphys as a distinct category [7]. Although MDAs mostly affect the female reproductive tract, developmental defects of the urinary system may also coexist due to the close embryologic relationship of mesonephric and paramesonephric ducts [2].

Diagnosis of Mullerian anomalies can be challenging as they remain clinically asymptomatic or present with nonspecific symptoms. Additionally, limitations of existing imaging techniques may contribute to missed diagnosis. Ultrasonography, for instance, is the most commonly used diagnostic tool during infertility evaluation and antenatal care [2]. But sometimes it may fail to identify anomalies such as uterus didelphys, particularly when anatomical abnormality is not specifically assessed. In such cases, there may be a delay in the diagnosis until pregnancy, delivery, or intraoperative evaluation.

We report a rare diagnostic case of uterus didelphys in a patient with long-standing infertility and an unexpected spontaneous conception. The anomaly remained undetected through multiple antenatal ultrasonographic examinations. It was first suspected clinically in the third trimester after the per-speculum identification of the longitudinal vaginal septum and the two cervices. The pregnancy was additionally complicated by severe oligohydramnios, breech presentation, and asymmetric fetal growth restriction.

## Case presentation

A 41-year-old female from Gujarat, India, presented with a history of primary infertility for 22 years. She reported a history of prior fertility treatment, including hormonal therapy approximately 7-8 years ago; however, specific details regarding the medications and treatment protocols were not available. There was no documented evaluation for uterine anomalies during prior infertility workup. Despite this, no successful conception occurred. At her initial presentation, the patient complained of intermittent per-vaginal blood spotting, which she initially attributed to light, irregular menstrual flow, along with lower abdominal enlargement. A transabdominal ultrasonography was done that diagnosed an unexpected, incidental pregnancy at approximately 20 weeks of gestation. Following the diagnosis, she received regular antenatal care. Multiple antenatal USG (Ultrasonography) were performed; however, none detected the underlying uterine anomaly.

Later, in the third trimester, the patient presented to a peripheral health center with complaints of lower abdominal pain, suggesting uterine activity. The ultrasonography showed severe oligohydramnios, breech presentation, and asymmetric intrauterine growth restriction (IUGR), with an estimated gestational age of 32 weeks 6 days and estimated fetal weight of 1962 ± 294.25 grams. The asymmetry was evidenced by a significant lag in abdominal circumference (26.75 cm; corresponding to 30 weeks 6 days) compared to the biparietal diameter (8.75 cm; corresponding to 35 weeks 2 days) and head circumference (29.52 cm; corresponding to 32 weeks 4 days). She was referred from the peripheral health center to the tertiary center for further management in view of the complications.

On admission at the tertiary center, clinical examination showed she was normotensive (BP: 108/70 mmHg; pulse: 86 beats/min) and afebrile. Per abdomen examination was performed by inspection and palpation, including Leopold’s maneuvers, and showed a gravid uterus at 32-34 weeks of gestation, breech presentation, and fetal heart sounds present. The fetal heart rate was 158 at baseline, with moderate variability and normal accelerations. Local vaginal and per speculum examination revealed two vaginal canals separated by a longitudinal vaginal septum, each leading into a distinct cervix, findings highly suspicious of an underlying Müllerian duct anomaly. Per vaginal examination demonstrated a closed cervical os in both the right and the left cervix, indicating absence of active labor, although uterine contractions were noted clinically (Figure 1). The patient was immediately booked for an Emergency LSCS (lower segment caesarean section) in view of advanced maternal age, severe oligohydramnios, asymmetric IUGR, and breech presentation.

**Figure 1:**
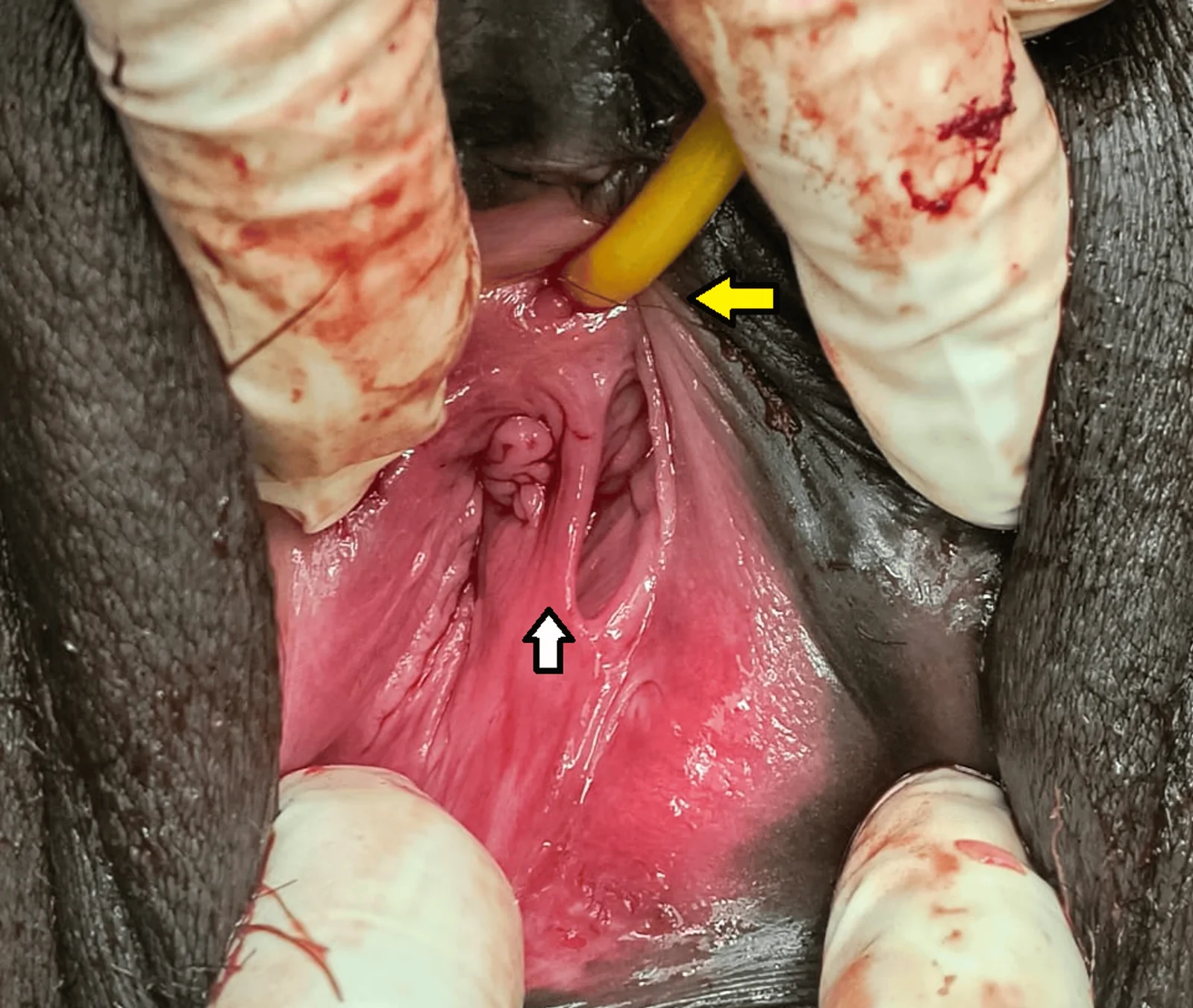
Local vaginal examination Local vaginal examination showing a longitudinal vaginal septum (white arrow) separating two vaginal canals, each leading to a distinct cervix, with the urethral meatus and Foley catheter in situ (yellow arrow). The identification of the vaginal septum and duplicated cervices raised clinical suspicion of an underlying Müllerian anomaly, subsequently confirmed intraoperatively as uterus didelphys bicollis.

Intraoperatively, the suspected congenital uterine anomaly was confirmed. The uterus was found to consist of two separate uterine horns that were not communicating, each leading into two distinct cervices, consistent with uterus didelphys bicollis. The pregnancy was located in the right uterine horn, which was enlarged corresponding to the period of gestation, while the contralateral horn was non-gravid and smaller in size (Figure 2). The placenta was anterior, grade 2, and no gross abnormalities were noted.

**Figure 2:**
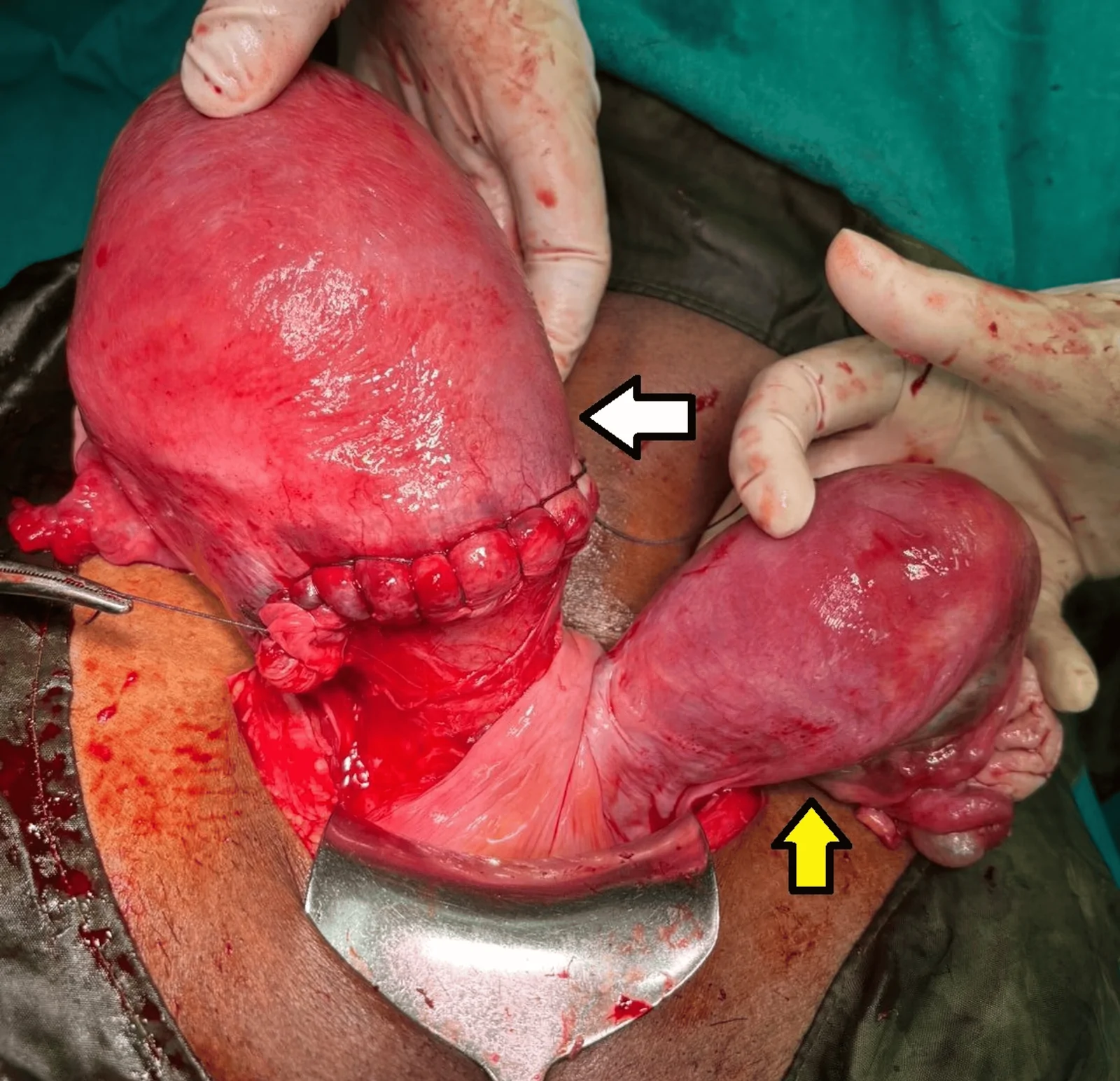
Intraoperative demonstration of uterus didelphys Intraoperative photograph showing complete uterine duplication, with pregnancy confined to the right uterine cavity. The right gravid uterus (white arrow) and separate left non-gravid uterus (yellow arrow) are visible, with a distinct longitudinal cleft containing intervening peritoneal tissue between the two uterine bodies, demonstrating complete uterine duplication.

The patient had a caesarean delivery of a live male baby weighing 1.810 kilograms. The Apgar scores were 6 and 8 at 1 minute and 5 minutes, respectively, with rapid improvement following routine neonatal support. The caesarean section was completed without any intraoperative complications, and no additional surgical intervention was performed on either uterine horn. The neonate required admission to the NICU (Neonatal Intensive Care Unit) to remain under observation owing to prematurity and antenatal complications.

The postoperative course was uneventful. The mother recovered well. A transabdominal ultrasound was done on postoperative day-3 to assess the renal and uterine anatomy. It revealed a distinct uterus didelphys with an involuting right gravid horn and an adjacent smaller left non-gravid horn with decidualized endometrium, and normal bilateral kidneys with no evidence of renal agenesis or hydronephrosis.

The newborn was discharged from NICU on day 7, and the mother was discharged on postoperative day 9, both in stable condition. The patient was informed and counseled regarding her diagnosis and related potential complications, given her advanced maternal age and her high-risk status for future pregnancies. The clinical course and key diagnostic and management events are chronologically summarized in Table 1.

**Table 1:** Chronological timeline of clinical events, diagnostics, and management USG, ultrasonography; IUGR, intrauterine growth restriction; LSCS, lower-segment cesarean section; NICU, neonatal intensive care unit

Timeline/Gestational Age	History/Clinical Presentation/Diagnostic Findings	Key Outcome/Action
Prior History (22 years)	Primary infertility; prior hormonal treatment of infertility (undocumented) 7-8 years ago	Spontaneous unassisted/natural conception
~20 weeks	Spotting, abdominal enlargement; USG-confirmed pregnancy	Initiated routine antenatal care
20-32 weeks	Routine serial antenatal ultrasounds	Diagnostic gap: uterine anomaly undetected; normal antenatal scans
32 weeks 6 days	Abdominal pain; USG: oligohydramnios, breech, asymmetric IUGR	Referred to a higher center
Admission to Tertiary Center	Speculum exam: 2 vaginal canals, longitudinal vaginal septum, 2 cervices	Suspected underlying Müllerian anomaly
	High risk status: due to advanced maternal age, breech, oligohydramnios, IUGR	Booked for Emergency LSCS
Intraoperative	Intraoperative visualization of two non-communicating uterine horns, pregnancy in right horn.	Confirmed uterus didelphys bicollis, delivered live male infant (1.81 kg), admitted to NICU
Postoperative Day 3	Transabdominal USG evaluating uterine and renal anatomy	Confirmed didelphys; normal bilateral kidneys (no renal agenesis/hydronephrosis)
Postoperative Day 7	Neonate- healthy, vitally stable	Discharged from NICU
Postoperative Day 9	Postoperative course uneventful, good recovery, mother vitally stable	Mother discharged with high risk pregnancy counselling

## Discussion

Uterus didelphys is a rare Müllerian duct anomaly resulting in two non-communicating uterine cavities and two cervices. In around 30% of cases, a longitudinal vaginal septum is also present [1]. Despite the anatomical defect, most women are asymptomatic, and fertility may be relatively preserved. Moreover, women with uterus didelphys had better clinical pregnancy rates (natural conception) as compared to other Müllerian anomalies (septate, arcuate, unicornuate uterus), although lower than those in women with a normal uterus [3]. However, reproductive outcomes remain variable, and infertility or the need for fertility treatment has been reported in women with uterus didelphys. Zhang et al. reported a greater need for infertility treatment among women with uterus didelphys than in those with other Müllerian anomalies, while Shah et al. described a case of uterus didelphys with cervical duplication presenting with infertility [8,9].

Pregnancy in uterus didelphys is associated with increased risk of developing obstetric complications like prematurity, fetal growth restriction, malpresentation, and cesarean delivery, which may occur potentially due to constrained space and suboptimal placentation. Heinonen et al., in their study of 49 cases of uterus didelphys, reported prematurity in 24%, fetal growth restriction in 11%, and cesarean delivery in 84% of the 71 pregnancies, with a fetal survival rate of 75% [6]. In a large population-based cohort study of 2,255 cases of uterus didelphys, Kadour et al. found higher adjusted odds of ​preterm premature rupture of membranes (PPROM), preterm delivery, and cesarean delivery as compared with other Müllerian anomalies [10]. The cases summarized in Table 2 further demonstrate this variable obstetric presentation associated with Müllerian anomalies, including malpresentation, fetal growth restriction, oligohydramnios, hypertensive disorders, abnormal labour, and fetal distress.

**Table 2:** Comparative review of reported cases of pregnancy in uterus didelphys comparing clinical presentations, diagnostic findings, and obstetric outcomes IUGR, intrauterine growth restriction; LSCS, lower-segment caesarean section; USG, ultrasonography; SAB, spontaneous abortion; PROM, premature rupture of membranes; SVD, spontaneous vaginal delivery;  IUGR, intrauterine growth restriction; CTG, cardiotocography.

Study	Age in Years/ G-P (Gravida-Parity)	Antenatal Recognition of Didelphys	Pregnancy/Obstetric Complication	Gestational Age at Delivery	Fetal Presentation	Mode of Delivery	Key Outcome/Intraoperative Finding
Present case	41/ G1P0	Not recognized	Advanced maternal age, severe oligohydramnios, breech, IUGR	32+6 weeks	Breech	Emergency LSCS	Intraoperative diagnosis; live male infant; pregnancy in right horn; non-gravid left horn; 2 cervices
Rezai et al., 2015 [12]	29/ G2P1	Suspected in 1^st^ pregnancy; USG suggested bicornuate vs didelphys; confirmed by USG in 2^nd^ pregnancy	Previous SAB; PROM at 38+4 weeks; chorioamnionitis	38+4 weeks	Vertex	Vaginal Delivery	Live healthy infant; complete vaginal septum tear during delivery; pregnancy in left horn of uterus
Sawai et al., 2017 [13]	23/ G1P0	Not recognized	Type IV placenta previa, severe oligohydramnios, severe IUGR	39+4 weeks	Oblique lie	Emergency LSCS	Intraoperative diagnosis; live female infant; pregnancy in left horn; non-gravid right horn; longitudinal vaginal septum with 2 cervices
Awolude et al., 2022 [14]	36/ G4P1+2 (1 SVD, 2 miscarriages)	Suspected antenatally; USG at 23 weeks showed two uterine horns	Two 1^st^ trimester pregnancy losses, labor in footling breech	38+4 weeks	Footling breech	Emergency LSCS	Intraoperative diagnosis; live infant; pregnancy in right horn; non-gravid left horn
Gaily et al., 2024 [15]	21/ G1P0	USG at 7 weeks: bicornuate uterus; longitudinal vaginal septum during attempted cerclage	Borderline cervical length (2.5 cm), longitudinal vaginal septum preventing cerclage placement	37 weeks	-	Elective LSCS	Intraoperative diagnosis; live infant
Pillai et al., 2025 [16]	27/ G5P4L3 (Previous 3 SVD, 1 LSCS with history of sterilization)	Not recognized	Severe preeclampsia	36+2 weeks	-	Emergency LSCS	Intraoperative diagnosis; pregnancy in left horn; non-gravid right horn; 2 cervices, single vagina
Shahid et al., 2025 [17]	21/ G1P0	Yes; identified on USG at 30 weeks 3 days & per-vaginal exam	Severe preeclampsia, breech presentation	36 weeks	Breech	Emergency LSCS	Pregnancy in right horn; non-gravid left horn; completely separate, non-communicating uteri
Alrassad et al., 2025 [18]	34/ G3P2	Not recognized	Prior 2 LSCS; active labour at 7 cm dilation with prior scar	-	Not specified (reassuring fetal status)	Emergency LSCS	Intraoperative diagnosis; pregnancy in left horn; non-gravid right uterus had well-healed scar from prior pregnancy
Al-Saad et al., 2026 [19]	20/ G1P0	Not recognized	Breech presentation	39 weeks	Breech	Elective LSCS	Intraoperative diagnosis; live male infant; 2^nd^ uterus identified; 2 cervices without vaginal septum
Roka et al., 2026 [20]	19/ G1P0	Not recognized	Fetal distress (persistent fetal bradycardia/CTG decelerations)	38 weeks	Cephalic	Emergency LSCS	Intraoperative diagnosis; live male infant; pregnancy in left horn; non-gravid right horn; 2 cervices

The diagnosis of a Müllerian anomaly relies on pelvic imaging methods, with two-dimensional ultrasonography (2D-USG) being the most commonly used initial modality. Routine antenatal 2D-USG focuses primarily on fetal assessment, and evaluation of maternal uterine morphology may be limited. Consequently, when a Müllerian anomaly is not suspected, it may fail to appreciate duplicated uterine cavities, fundal anatomy, or any other associated cervical and vaginal abnormalities. Pelvic imaging techniques such as three-dimensional ultrasonography (3D-USG) and magnetic resonance imaging (MRI) provide comprehensive assessment of the pelvic anatomy, with high sensitivity and specificity for diagnosing Müllerian anomalies [2]. A review by Jayaprakasan et al. reported that 3D-USG is the gold standard for diagnosing and assessing uterine anomalies as it allows simultaneous visualization of the internal endometrial cavity and external serosal contour of the uterine fundus through its coronal plane [11]. An MRI provides multiplanar imaging, superior soft-tissue characterization, and a wider field of assessment. It can also help evaluate the presence of ovaries and associated renal malformations, and is particularly useful when complex Müllerian anomalies are suspected or when findings from other imaging modalities are inconclusive [2,11]. Despite the high diagnostic accuracy of these advanced modalities, their clinical utility depends on appropriate clinical suspicion and availability. Several reported cases were diagnosed only during a cesarean delivery, whereas others were either recognized antenatally or initially interpreted as bicornuate uteri. Table 2 also shows a detailed comparison of such reported cases with the present case.

This highlights that the diagnostic challenge is not only imaging limitations, but also the failure to assess uterine morphology when clinical suspicion is low. Although the reported patient had long-standing primary infertility, the absence of prior infertility evaluation records limits assessment of whether earlier diagnosis was possible. This case also emphasizes the importance of local per- speculum or per-vaginal examinations. Identification of a longitudinal vaginal septum or duplicated cervices may raise suspicion of an underlying anomaly and prompt further evaluation with appropriate imaging.

## Conclusions

Uterus didelphys may remain asymptomatic and undiagnosed despite long-standing infertility and repeated antenatal imaging. A thorough pelvic examination, including assessment for a longitudinal septum and duplicated cervices, may provide an important clinical clue and prompt for targeted imaging. Although the history available does not establish whether the anomaly contributed to this prolonged period of infertility or whether earlier diagnosis was possible, recognition during an earlier evaluation could have facilitated more timely diagnostic counselling. Early diagnosis of Müllerian anomalies may support individualized counselling, appropriate imaging, and risk-based obstetric surveillance.
